# Batch correction and harmonization of –Omics datasets with a tunable median polish of ratio

**DOI:** 10.3389/fsysb.2023.1092341

**Published:** 2023-04-12

**Authors:** Eric B. Dammer, Nicholas T. Seyfried, Erik C. B. Johnson

**Affiliations:** ^1^ Goizueta Alzheimer’s Disease Research Center, Emory University School of Medicine, Atlanta, GA, United States; ^2^ Department of Biochemistry, Emory University School of Medicine, Atlanta, GA, United States; ^3^ Department of Neurology, Emory University School of Medicine, Atlanta, GA, United States

**Keywords:** batch correction, multi-platform, multi-cohort, proteomics, median polish, multi-omics, TAMPOR

## Abstract

Large scale −omics datasets can provide new insights into normal and disease-related biology when analyzed through a systems biology framework. However, technical artefacts present in most −omics datasets due to variations in sample preparation, batching, platform settings, personnel, and other experimental procedures prevent useful analyses of such data without prior adjustment for these technical factors. Here, we demonstrate a tunable median polish of ratio (TAMPOR) approach for batch effect correction and agglomeration of multiple, multi-batch, site-specific cohorts into a single analyte abundance data matrix that is suitable for systems biology analyses. We illustrate the utility and versatility of TAMPOR through four distinct use cases where the method has been applied to different proteomic datasets, some of which contain a specific defect that must be addressed prior to analysis. We compare quality control metrics and sources of variance before and after application of TAMPOR to show that TAMPOR is effective at removing batch effects and other unwanted sources of variance in −omics data. We also show how TAMPOR can be used to harmonize −omics datasets even when the data are acquired using different analytical approaches. TAMPOR is a powerful and flexible approach for cleaning and harmonization of −omics data prior to downstream systems biology analysis.

## Introduction

In Alzheimer’s disease (AD) research, quantitative large-scale analysis of RNA transcripts, proteins, and metabolites in brain tissue and biofluids has become a powerful approach to increase understanding of the complex molecular alterations that characterize this increasingly prevalent disease (De Jager et al., 2018; Ma et al., 2020; Neff et al., 2021; Wörheide et al., 2021). Individual analytes within each −omic dataset now often number in the many thousands, providing a rich snapshot of biological states at the time of analysis. Integration across multiple different types of −omics datasets holds promise to further increase our understanding of complex molecular networks and responses in disease states such as AD (De Jager et al., 2018; Ma et al., 2020; Johnson et al., 2022). However, the ability to gain useful information and insight from such individual and multi-omic datasets fundamentally rests on the biological signal present within each dataset. Unfortunately, most −omics studies and datasets suffer from significant nuisance batch artefacts that mask the underlying biological signals of interest. Furthermore, integration of separate datasets that are not designed *a priori* for integration presents a significant challenge to data harmonization. Therefore, separate analyses specific to each dataset are often employed, with meta-analysis used to assess consistency of findings across individual studies (Haytural et al., 2021), but without the benefit of the full power of all samples harmonized and analyzed as one dataset when inter-dataset systematic differences such as tissue type or region are not a focus of the analysis. Here, we describe a tunable median polish of ratio (TAMPOR) method to minimize nuisance batch effects and harmonize separate datasets for subsequent analyses. We focus on the applications of TAMPOR as applied to proteomic data, but in theory the method is applicable to any −omic dataset. We first describe the TAMPOR method, and then illustrate through four use-case scenarios how TAMPOR has been applied to solve problems of batch and dataset harmonization.

## Results

### Description of the TAMPOR algorithm and its visualization

John Tukey first described the median polish approach for exploratory data analysis in 1977 (Tukey, 1977). As previously published we adapted its implementation in TAMPOR (Higginbotham et al., 2020; Johnson et al., 2020; Dill et al., 2021; Johnson et al., 2022) where the equation general form for rowwise ratio calculation in TAMPOR is expressed for each row ***i***, each sample ***j***, and each batch ***k***_***(1:n)***_ over ***n*** batches as follows.

$$\begin{array}{c} {ratio}_{{ij}_{\left(k_{n}\right)}}=\frac{{{abundance}_{ij}}_{\left(k_{n}\right)}}{median\left({abundances}_{i}\;\epsilon \;j_{\left(k_{n}\right)}\right)}\cdot \frac{grand\;median\left\{M_{\left(k_{1}\right)},M_{\left(k_{2}\right)},...M_{\left(k_{n}\right)}\right\}}{M_{\left(k_{n}\right)}}\\ where\;M_{\left(k_{n}\right)}=median\left(\frac{{abundances}_{i}\;\epsilon \;j_{\left(k_{n}\right)}}{median\left({abundances}_{i}\;\epsilon \;j_{\left(k_{n}\right)}\right)}\right) \end{array}$$ (1)

The above equation leverages the central tendency of abundance within a protein (each row of the abundance matrix). If there are standard replicate samples in every batch, here termed global internal standard (GIS), the equation may be tuned so that each of the two terms’ denominators can leverage the “all sample physical average” of the GIS as the enforced central tendency of abundance within a protein (row). The GIS thus serve as bridging samples (Ping et al., 2018). Such tuned versions of TAMPOR, or modes of the general TAMPOR equation (Eq. 1), are described in use cases below. Even if no sample replicates are used as bridging samples, by correcting batches which have been designed to contain random subsets of samples with balanced biological traits of interest in each batch, the above equation applies verbatim for removal of batch effects using all samples in each batch. This use case has been recently demonstrated by others (Modekurty, 2020) as well as ourselves (Johnson et al., 2020). The tunability of TAMPOR improves its applicability to more datasets, and which mode is employed in practice depends on the availability of GIS in all batches of a dataset, or pre-set balancing and randomization of samples by important traits that should not be confounded with batch, particularly when no GIS samples are employed. We provide a walkthrough demonstration and simplified code for a GIS-tuned version of TAMPOR at https://github.com/edammer/TAMPOR/blob/master/walkthrough.md.

**FIGURE 1:**
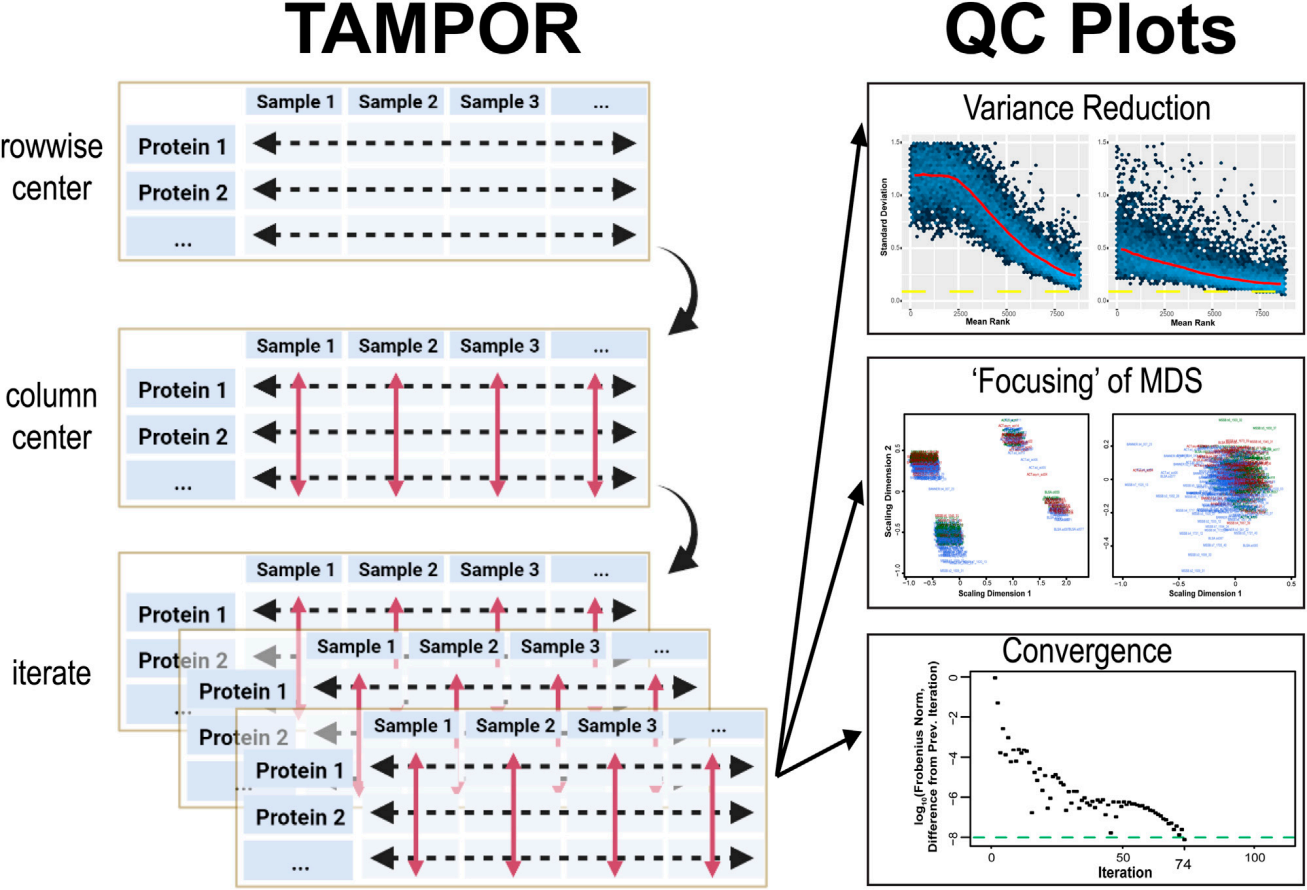
TAMPOR for median polish of abundance and quality control (QC) visualization of correction of batch effect. *Left*, TAMPOR algorithm implements a 2-step median polish, first calculating a ratio for each abundance value using a two-term equation operating on rows to bring the data in different batches towards a common denominator (“row-wise centering”). Then, log_2_ (ratio) data are centered at a median value of 0 (red arrows, “column centering”). The initial median abundance of a row is then multiplied by all antilogged ratios within that row, this is repeated for all rows to reproduce data in the same format as input, and the 2-way median centering process is iterated until convergence. *Right*, visualization of quality control is performed in 3 ways by the TAMPOR function in R. Mean-standard deviation (mean-SD) plots should show a reduction in overall variance for the entire population of proteins ranked by their mean abundance, from high to low. Multidimensional scaling (MDS) plots show a focusing of variance from multiple foci representing batches, to a single focus, indicating resolution of the batch effect. Last, the convergence criterion, difference in Frobenius norm, is tracked over successive iterations of the algorithm. The algorithm by default runs for up to 250 iterations, or until convergence is reached (a difference in the Frobenius norm from the previous iteration of <10^–8^).

After driving each set of values row by row towards a distribution with a center at the central tendency for the row (a calculation of ratio tending towards 1), the ratio matrix is log_2_-transformed, and each sample (column) has its median value subtracted, centering sample-wise median or central tendency at a log_2_ ratio of 0. Then data are anti-logged, each row’s values are multiplied by the row-wise (protein) median abundances saved from before ratio calculation, and the process is iterated (Figure 1).

QC of TAMPOR output is provided through three built-in visualizations of the starting data, after a single application of row-wise ratio, and after convergence or the final iteration. A robust convergence criterion stops iterations when a matrix statistic no longer changes appreciably (at the iteration when difference from the previous iteration’s Frobenius norm is less than 10^–8^). This convergence threshold was chosen from historic literature, e.g., (Bar-itzhack and Fegley, 1969), and manuscripts citing this reference. We appreciate that not all datasets achieve convergence by standard median polish (Fink, 1988); when this occurs, a user can consider such a TAMPOR run as preliminary, and judge from the QC plots where precipitous drop-offs towards convergence cease as a reasonable maximum number of iterations to run in a final TAMPOR pass. In addition to a plot tracking the decreasing trend in difference between successive iterations’ Frobenius norms, the user can expect to see a successful run show a more “focused” two-dimensional multi-dimensional scaling (MDS) plot in which the distribution of points representing inter-sample variance becomes a single “shotgun blast,” where it may start as multiple such foci representing batches of samples. Overall variance reduction is visualized in comparison of mean-standard deviation (SD) plots of the log_2_ abundance data before TAMPOR, after naïve ratio calculation, and after TAMPOR.

Running TAMPOR, a user will generally observe a fast early approach towards the convergence criterion, whether it is reached or not. Second, the user should observe a decrease in SD across all ranks of proteins in mean-SD plots after naïve ratio correction, followed by a further decrease with TAMPOR, if it is more effective than simple ratio over GIS. Third, MDS of samples colored by batch loses distinct clustering by batch. If any of these expectations are not visualized, one should check or possibly change how TAMPOR is applied to the data input.

### Assumptions of TAMPOR compared to other batch correction strategies

The primary assumption of TAMPOR is that, by adjusting the central tendencies of batches of the abundance matrix towards equality, followed by sample-wise central tendency centering at a log2 (ratio) of zero, and iterating, the majority of variance due to either batching or unequal sample loading will be mitigated. Note that an iteration of TAMPOR completes through a second step in the median polish algorithm to equalize the median or central tendencies by sample, not assuming each sample has equal overall analyte concentration, but that after correction is complete, any such loading disparities are corrected. This second step of TAMPOR leverages the wide variance distribution (i.e., dynamic range over orders of magnitude) in −omics data between different gene products in each individual sample; this variance, in general, is greater than intra-protein variance across samples. Certain scaling of data, where gross differences between different protein abundances within samples have been ablated, is not appropriate for TAMPOR because the centering of all such values within a sample is not reliable, with median polish of such data having no meaningful sample-wise variance to find a central tendency. An example of such scaling would be setting each row’s (protein’s) values to a fraction of the maximum value, with the maximum at 100 (%) on each row.

A second assumption is that missing abundance data occurs without extreme bias so that the median of the distribution of log_2_ (abundance ratios) in any subset represents a true central tendency (i.e., the median datapoint is a true, non-noise value). Thus, missing data in the matrix is limited to less than 50 percent in standard applications of TAMPOR. Leveraging a ratio approach in the median polish using log_2_-transformed ratios iteratively drives the central tendency of each group of samples towards a log_2_-ratio of 0, and unlogged, the central tendency approaches a ratio of 1:1. This is true either for the population of all samples in the case that a GIS is used, or if a subset of biological replicates (e.g., controls) are used in place of a GIS, then the ratio drives towards 1:1 for the subset of samples that are used as denominators in the polish of ratio. The matrix at the end of the process has equalized central tendencies not only for every row (protein) but also every sample, which also corrects for unequal loading, where benchtop methods and less robust normalization often intend gross equal loading of protein wet weight or total RNA, but do so imperfectly. Thus, downstream analysis of TAMPOR abundance should assume equal loading of each sample by a common total protein weight. Any systematic concentration difference, e.g., by diagnosis, will not be preserved or detectible; if the researcher wants to maintain differences in sample loading, e.g., by biofluid volume, then TAMPOR is not the ideal normalization algorithm. In practice, TAMPOR can follow and use unlogged abundance data that underwent a simple normalization, such as scaling of sample summed protein signal, if the normalization function maintains the dynamic range of measurements between proteins [e.g., the “normalized abundance” output from the mass spectrometry Proteome Discoverer software suite (ThermoFisher, 2017)]. This is not equivalent to the effect of iterative leveraging of sample-wise central tendency in TAMPOR, however. Note that it remains untested whether batches of abundance data with missingness not at random (MNAR), e.g., LFQ proteomics, could be successfully harmonized with data having more missingness at random, or batchwise missingness, like that of TMT batched datasets; however, given appropriate missingness thresholds of less than 50 percent, if not more stringent, such harmonization should not be improbable.

The variance structure described above for proteomic abundance data is also found in transcriptomic abundance data expressed as read counts, fragments per kilobase per million mapped reads (FPKM), or transcripts per million (TPM) This data generally has zero and low values rather than missing values. However, transcriptomic data often has a bimodal distribution of log_2_ (abundance), with the lower mode (and especially the left tail) representing noise. To overcome this bias in central tendency, the rows of data can be censored, treating zero and low signal (mostly noise) values as missing data and setting a maximum noise-level count threshold for each row (gene product), throwing out rows with too many noise values and keeping only rows that are well expressed in the biological matrix (Dill et al., 2021). Following such left-tail censoring, such data are amenable to the same TAMPOR-based correction approach as proteomic data. In the case of RNA-Seq, whether TPM, FPKM, normalized or integer counts, less data is more; an added benefit is that the matrix of sometimes 60,000 gene products can be collapsed to under 20,000, which is typically the true number of non-noise transcripts represented in any single tissue, tumor, or cell type.

Other common batch correction techniques have different assumptions. For example, Bayesian inference, as employed in the ComBat algorithm, relies on common sample replicates (technical or biological) in batches to infer and correct batchwise differences (Johnson et al., 2007). ComBat has been used to successfully remove multiple overlaid batch effects from proteomics data (Seyfried et al., 2017), even scaled abundance data stripped of interprotein dynamic range (Gutierrez-Quiceno et al., 2021). However, ComBat cannot tolerate missing data, as in the microarray data to which it was originally applied. Consequently for proteomics data, this requirement can necessitate at least temporary imputation of missing data points (Gutierrez-Quiceno et al., 2021); when data are TMT batches, K-nearest neighbor imputation is appropriate; and when data represent LFQ batches, left tail distribution imputation can be used. After correction, missing values can also be returned to the data. In the above publication leveraging ComBat, only 6,700 proteins with no missing values across 10 TMT batches were considered to avoid imputation of missing data points. We have recently recorrected the same underlying data with TAMPOR, thereby rescuing over 2,400 additional protein measurements missing in 5 or fewer batches, after multiplying scaled abundances by their protein-specific median abundance. The batch- and region-corrected data and comparisons of effect sizes for advanced chronic traumatic encephalopathy (CTE) versus control samples in this data are available from https://www.synapse.org/EmoryCTE.

Regression, which leverages a linear fit of central tendency, essentially adjusts the slope of the fit line to 0 by subtracting the component of variance from data that coincides with that slope. Regression-based batch correction has the benefit of being able to adjust multiple factors at once using a multivariate model. However, the regression model’s terms may interact, which complicates choosing an appropriate model. Surrogate variable analysis (SVA) is a commonly used regression-based method which can remove unknown or latent variables’ correlation not affecting variance across the known sample groups (Leek et al., 2012). These latent variables may represent meaningful biological variability required for the open, unbiased, data exploration inherent in systems biology analysis (Jaffe et al., 2015); thus, SVA can be over-aggressive in correcting artefactual biases, particularly when the regressed data is intended for consideration in combination with other datasets where different latent variables likely hide. Notably, both regression-based batch correction of multiple factors and sequential application of TAMPOR can handle multiple overlaid batch effects.

Nonetheless, given the similarities of all batch correction methods and their limitations, good experiment or cohort design dictates a randomization of samples into batches that are balanced for all sample traits, following the general rule that all batches should contain some of each categorical sample (e.g., mixed sex when both are in a cohort, a mixture of diagnoses, common ranges for continuous variable traits, etc.), thereby avoiding confounds with batch in downstream analysis. To the extent these principles are violated along with the assumption of independence of batch and analyte signals across all samples of a dataset, all additive model batch correction methods including TAMPOR cannot reliably adjust for batch without affecting or biasing signal. However, TAMPOR tuned to use only GIS for denominators, with GIS sample(s) in every batch, and where all GIS (pooled mixture replicate) samples are not outliers, could overcome the above strong point.

### Use cases of TAMPOR for batch correction and harmonization of published datasets

In the simplest batch correction scheme, intra-batch GIS sample replicates are usually leveraged as a denominator of the other intra-batch abundances, and all ratios of abundance/GIS_intrabatch_ are then considered as batch corrected (Ping et al., 2018). Although this simple ratio is often considered as sufficient batch correction, our first use case of TAMPOR demonstrates this assumption to be naïve.

### Use case 1: When technical replicates used for batch correction are outliers

**FIGURE 2:**
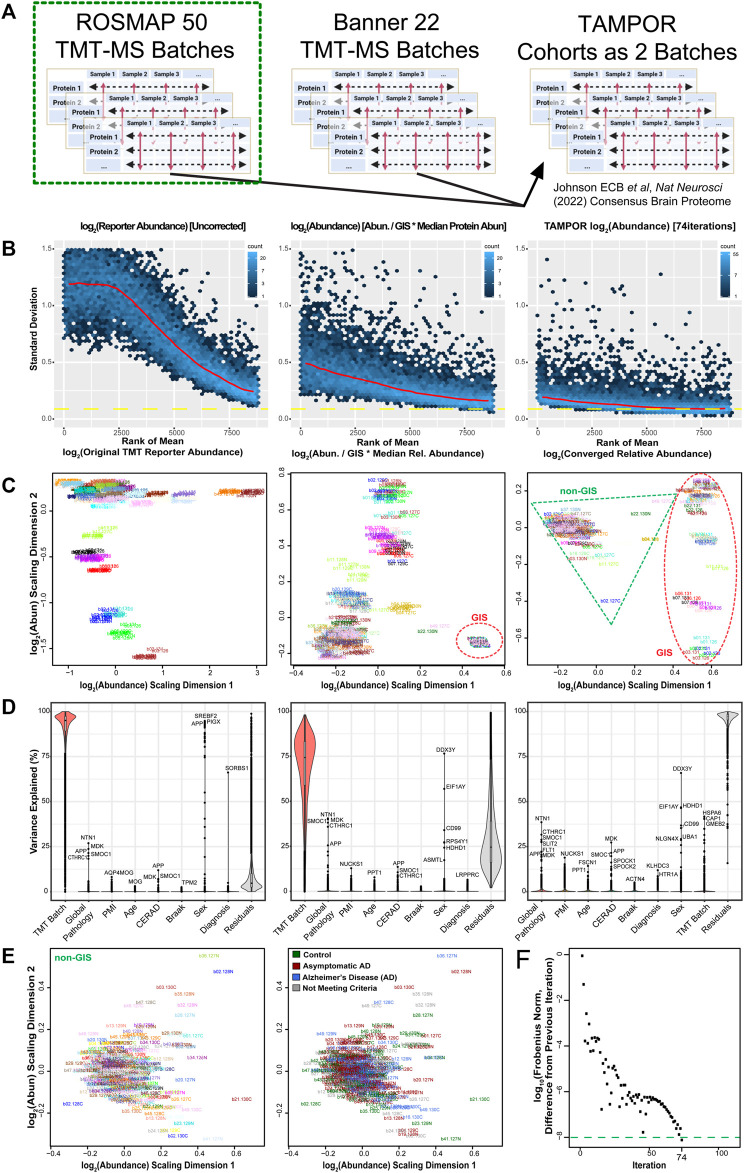
Use case 1: TAMPOR correction of TMT-MS data of 400 frontal cortex brain proteomes with a defect in GIS. **(A)** TAMPOR was performed sequentially on two cohorts of TMT-MS data, first to remove intracohort batch effects, and then to harmonize across the two cohorts, ROSMAP (50 TMT-MS batches), and Banner-SunHealth (22 TMT-MS batches). **(B)** Mean-SD plots of variance in original ROSMAP50 TMT log_2_ abundance data for 400 case samples plus 100 GIS samples (*left*), naïve ratio-corrected log_2_ abundance (*center*), and TAMPOR log_2_ abundance (*right*), indicating progressive improvement in reduction of overall variance. **(C)** MDS plots of inter-sample variance with sample names as labels, colored by batch. *Left*, original ROSMAP50 TMT log_2_ abundance; *center*, naïve ratio-corrected log_2_ abundance; and *right*, TAMPOR log_2_ abundance. GIS sample cluster(s) are circled in the center and right panels with a red dashed ellipse. Non-GIS samples are within the green dashed triangle on the right. **(D)** Variance partitioning violin plots indicating median variance explained by each variable, with the top proteins for clinical, pathological, and demographic variables specified. *Left*, original ROSMAP50 TMT log_2_ abundance; *center*, naïve ratio-corrected log_2_ abundance; and *right*, TAMPOR log_2_ abundance. **(E)** *Left*, The non-GIS samples in the rightmost panel C are replotted in their own MDS of samples colored by batch. *Right*, sample labels are recolored by their case diagnoses at time of death. **(F)** Convergence plot from TAMPOR for the ROSMAP 50 batch TMT abundance data. TAMPOR abundance converged in 74 iterations.

Fifty batches of TMT-labeled peptide digests of total homogenate proteins from dorsolateral prefrontal cortex of human donors were fractionated offline and then fractions were analyzed by LC-TMT-MS/MS in a cohort of 400 case samples as previously described (Johnson et al., 2020; Johnson et al., 2022). Eight case samples plus two GIS replicates (all sample equal mixture) were in each batch with 10 total channels per batch. In this large experiment, GIS replicate digestion was performed separately from digestion of the multiplex batch mixtures, which decreased the utility of the GIS for normalization as a simple ratio denominator—not removing batch effects effectively at all, as described below. These sample abundances were ultimately intended for inclusion as part of a larger 2-cohort dataset, which was harmonized after intracohort batch correction also using TAMPOR (Johnson et al., 2022). In this use case we focus on the first use of TAMPOR, which was to correct for batch within the 400 case ROSMAP cohort (Figure 2A). This use case demonstrates that a tuned GIS-plus-non-GIS based denominator set for the TAMPOR equation affords substantial improvement versus GIS-only as a denominator when GIS samples alone are poor bridging samples.

Upon performing naïve ratio of abundance/GIS_intrabatch_ mean, overall variance of the data was reduced (Figure 2B, middle vs. left panel), but batch effects were still evident as multiple foci relating to groups of batches in the MDS plot comparison (Figure 2C, middle vs. left panel). Variance partitioning of all 8,817 protein isoforms in the matrix with less than 50 percent missing abundances per protein highlights that the median protein still had over 74 percent of variance explained by batch, down from 95 percent (Figure 2D, middle vs. left panel). TAMPOR was performed, using the tuning parameter useAllNonGIS = TRUE, which specifies the denominators in the more general Eq. 1 as

$$\begin{array}{c} {ratio}_{{ij}_{\left(k_{n}\right)}}=\frac{{{abundance}_{ij}}_{\left(k_{n}\right)}}{median\left(GIS\;{abundances}_{i}\;\epsilon \;j_{\left(k_{n}\right)}\right)}\;\cdot \;\frac{grand\;median\left\{M_{\left(k_{1}\right)},M_{\left(k_{2}\right)},...M_{\left(k_{n}\right)}\right\}}{M_{\left(k_{n}\right)}}\\ where\;M_{\left(k_{n}\right)}=median\left(\frac{non-GIS\;{abundances}_{i}\;\epsilon \;j_{\left(k_{n}\right)}}{median\left(GIS{abundances}_{i}\;\epsilon \;j_{\left(k_{n}\right)}\right)}\right) \end{array}$$ (2)

TAMPOR abundances showed further significant variance reduction, a single focal cluster of non-GIS samples in MDS, and median protein variance explained by TMT batch decreased to zero percent (Figures 2B–D, right panels). Interestingly, the GIS samples in MDS were now clearly appearing as separate clusters of outliers in MDS dimension 1, in contrast to their single cluster with naïve ratio. This demonstrates that the TAMPOR tuned to allow non-GIS abundances allows a converged result in which the true heterogeneity of the GIS samples, due to differential digestion *via* different enzyme batch and timing, does not impact the focusing of variance among the non-GIS samples. Proteins associated most strongly to the global pathology, CERAD, and Braak scores remained or strengthened in their association when comparing variance partitioning between naïve ratio or TAMPOR (Figure 2D, middle vs. right panel). Replotting MDS on the 400 non-GIS samples used for variance partition, batch specificity of samples does not contribute to subclusters of samples (colors representing different batches are scattered) (Figure 2E, left panel). Recoloring the samples in this MDS plot reveals that the most variant proteins are not driving gross differences between diagnosis groups of the cases, which due to their recruitment from a prospective community cohort, represent the continuum of disease progression over age.

The TAMPOR abundance matrix converged in 74 iterations (Figure 2F). This use case demonstrates TAMPOR as more versatile and robust than naïve abundance/GIS_intrabatch_ ratio for batch effect removal in data where all-sample average (GIS) replicates are included but may have defects.

### Use case 2: Harmonization of tissue collection site for multicohort analysis

**FIGURE 3:**
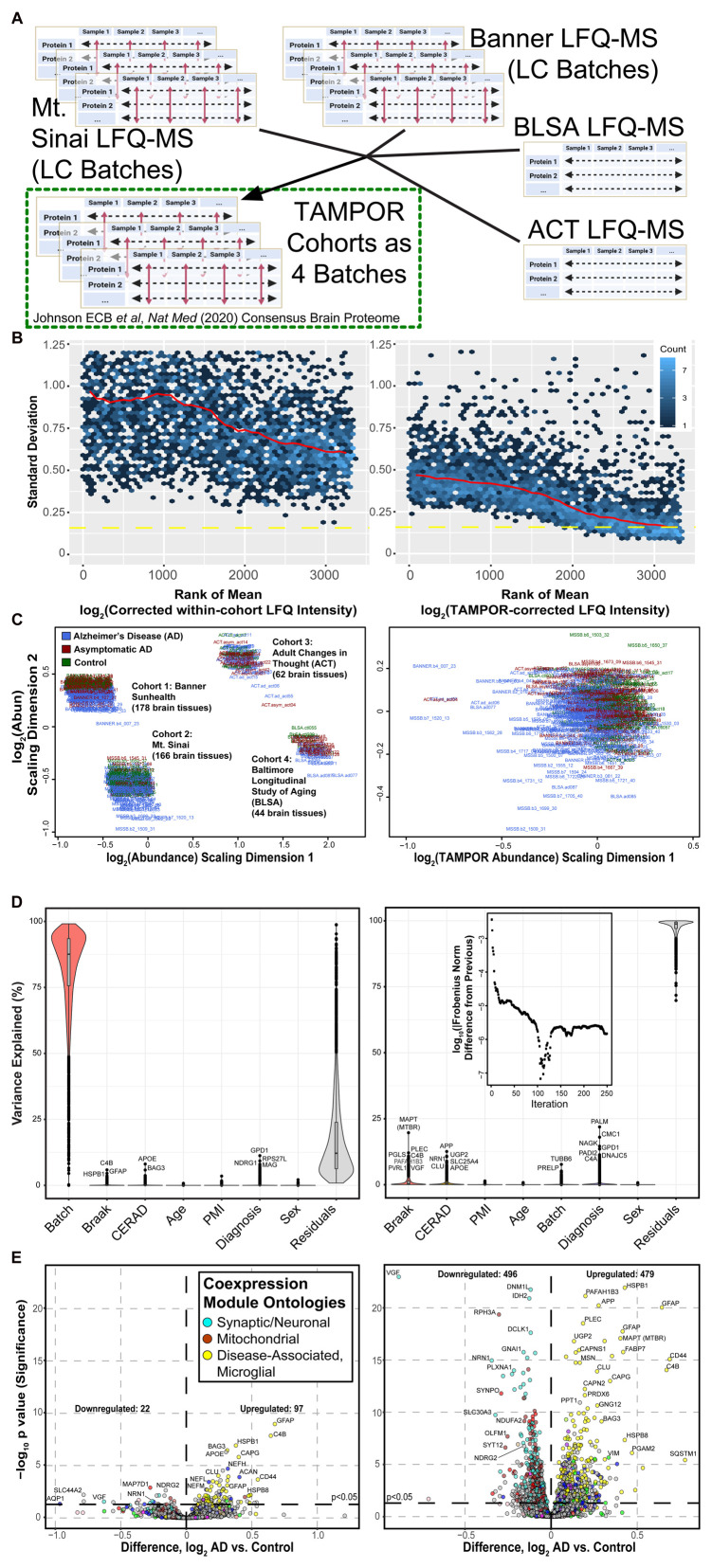
Use case 2: TAMPOR correction of multi-cohort LFQ-MS data for 450 prefrontal cortex tissue proteome case samples. **(A)** Four cohorts of LFQ-MS data collected over 3–4 years were batch-corrected and regressed before the multi-cohort abundance data could be harmonized with TAMPOR, addressing the site and inter-cohort variance as a batch effect. **(B)** Variance of all ranked proteins decreased comparing the mean-SD plot before TAMPOR, *left*, to after TAMPOR, *right*. **(C)** MDS of the 4 cohort log_2_ LFQ abundances before TAMPOR, *left*, and after, *right*. **(D)** Variance partition analysis of the same data before (*left*) and after TAMPOR (*right*). Inset shows the convergence plot for TAMPOR performing inter-cohort harmonization. **(E)** Volcano plot of differentially abundant proteins for AD versus control case samples among the 419 samples determined as final non-outlier case samples in the same data before (*left*) and after TAMPOR (*right*).

Label-free quantitation mass spectrometry (LFQ-MS) protein abundances from analysis of “single-shot” fraction injections on the same mass spectrometer were available for 4 cohorts of dorsolateral prefrontal cortex total homogenate (brain proteomes of Mt. Sinai, Banner-SunHealth, Baltimore Longitudinal Study of Aging/BLSA, and Adult Changes in Thought/ACT study participant-donors) (Figure 3A) as previously described (Johnson et al., 2020). The mass spectrometry analyses occurred over the course of 3 years, and were subject to mass spectrometer tuning, multiplier replacement, and different nano-flow liquid chromatography (LC) parameters and columns, posing a challenge for analysis as a single data set. These cohorts included cases that were selected to fit clearly into 3 diagnosis categories: non-cognitively impaired controls, asymptomatic AD, and symptomatic AD (Johnson et al., 2020). Two of the cohorts, Mt. Sinai and Banner, were themselves collected in multiple batches many with different LC columns and machine calibrations occurring over the course of the analyses. LFQ intensities of these cohorts were corrected using TAMPOR with global pooled reference samples serving as external standards, which were run at the beginning, middle, and end of each LC batch (Johnson et al., 2020). Age, sex, and postmortem interval covariance was removed by bootstrap regression in each cohort-specific log_2_ (abundance) matrix, and regression of the ACT cohort also considered a difference in white matter inclusion. Then, TAMPOR was run on the full data set of 450 case samples and 3,337 protein isoforms using the general, untuned, Eq. 1, in which denominators use all samples for central tendency (Figure 3A). This mode of TAMPOR is implemented with the parameter noGIS = TRUE.

Tissue collection site effects contributed to elevated variance in the 4-cohort data, since the mean-SD plot trendline for all proteins was lower after TAMPOR (Figure 3B), and the MDS visualized 4 distinct foci, which were reduced to one after TAMPOR (Figure 3C). Pathological and demographic trait correlations to each of the proteins in the data are shown as violin plots in Figure 3D, with each modeled trait sorted from left to right in order of decreasing median protein variance explained for the uncorrected input data (left panel) and the TAMPOR abundance output (right panel). Whereas the plaque pathology-associated CERAD score correlated best to APOE before TAMPOR, after TAMPOR the main protein component of plaques, APP (representing amyloid-β), reached the top-ranked protein for variance explained by CERAD score. Likewise, the aggregating portion of Tau protein, its microtubule binding repeat region that was considered as a separate protein (MAPT MTBR) and which gauges the scale of Tau-containing tangle spread in the brain (Dai et al., 2018; Johnson et al., 2020), appears at the top of the violin for Braak score. TAMPOR of this data did not reach convergence in the default maximum 250 iterations (Figure 3D, inset at top of right panel). Notably, postmortem interval (PMI), age, and sex, which were traits regressed for before the final TAMPOR pass harmonizing the four cohorts, remained uncorrelated to any protein after this TAMPOR pass consistent with the design of TAMPOR to introduce no bias and to maintain biological variance within provided input. Cohort site effects are ablated by TAMPOR, seen as the stark change in the violin area and height labeled “Batch.” Differentially abundant proteins viewed as a volcano plot comparing the AD to the control diagnosis group before and after TAMPOR (Figure 3E, left and right panels, respectively) reveal greatly augmented power to detect changed proteins, and proteins already meeting the nominal significance threshold before TAMPOR invariably improve in their significance after TAMPOR. This use case demonstrates that TAMPOR harmonization of multicohort abundance data for a unified single analysis unleashes statistical power of analysis by leveraging all available samples, and can be performed without a common GIS present in each cohort.

### Use case 3. Gauging batch correction precision with technical replicate samples

To benchmark the precision of TAMPOR compared to ratio of abundance/GIS_intrabatch_ in a TMT-labeled data set, we analyzed raw data from the published study by Dayon et al. (2018) which incorporates 120 case samples of cerebrospinal fluid (CSF) proteome-derived peptides, each replicated in 2 batches. Each batch has 2 GIS sample replicates. As the data were previously published using naïve ratio normalization from an independent search and quantification analysis, we reproduced the identification and quantification analysis from raw data, and compared naïve ratio to TAMPOR abundance, in which the least aggressive TAMPOR equation set was used:

$$\begin{array}{c} {ratio}_{{ij}_{\left(k_{n}\right)}}=\frac{{{abundance}_{ij}}_{\left(k_{n}\right)}}{median\left(GIS\;{abundances}_{i}\;\epsilon \;j_{\left(k_{n}\right)}\right)}\;\cdot \;\frac{grand\;median\left\{M_{\left(k_{1}\right)},M_{\left(k_{2}\right)},...M_{\left(k_{n}\right)}\right\}}{M_{\left(k_{n}\right)}}\\ where\;M_{\left(k_{n}\right)}=median\left(\frac{GIS\;{abundances}_{i}\;\epsilon \;j_{\left(k_{n}\right)}}{median\left(GIS{abundances}_{i}\;\epsilon \;j_{\left(k_{n}\right)}\right)}\right) \end{array}$$ (3)

**FIGURE 4:**
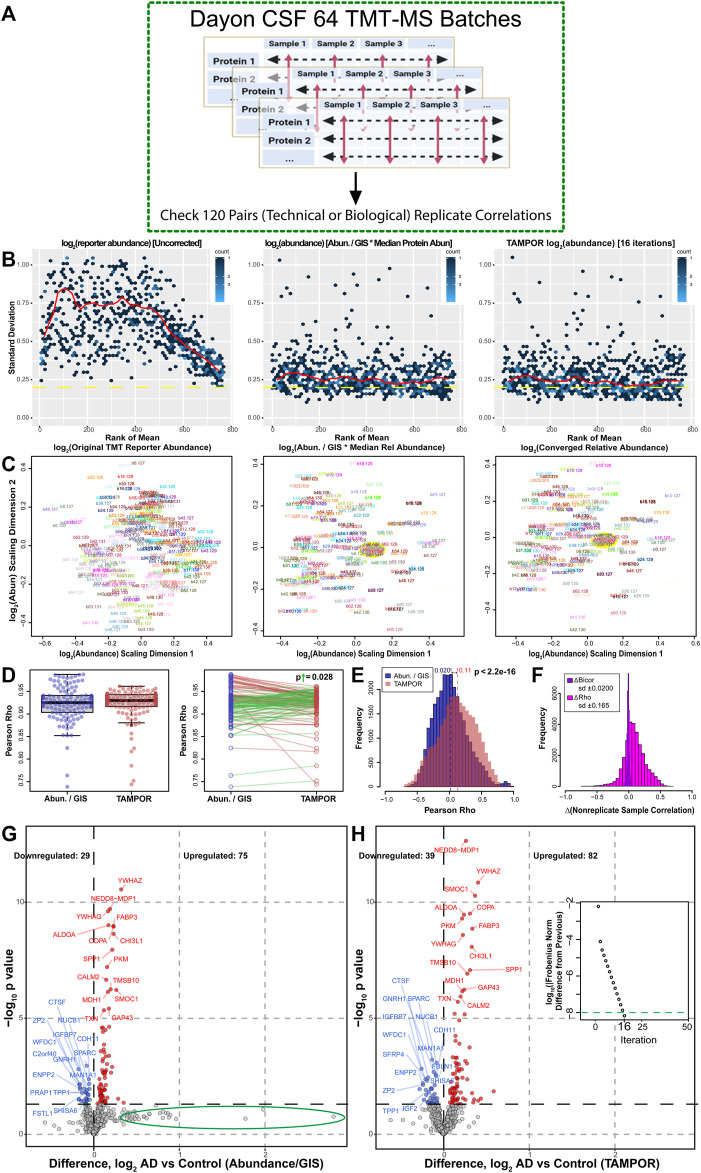
Use case 3: Comparison of TAMPOR versus naïve ratio of 64 batch TMT-MS CSF proteomics. **(A)** One hundred and twenty case samples of CSF were analyzed by TMT-MS, each with a technical replicate in a different batch loaded equally by volume as published in (Dayon et al., 2018). We reanalyzed the publicly available raw data and performed a head-to-head comparison of naïve ratio of abundance/GIS_intrabatch_ to TAMPOR abundance, paying special attention to replicate and non-replicate correlations in the data. **(B)** Mean-SD plots show variance in log_2_ TMT abundance without ratio-based correction (*left*), with naïve ratio (*center*), and with TAMPOR (*right*). **(C)** MDS plots show changes in intersample variance from uncorrected TMT log_2_ abundance (*left*), to naïve ratio-corrected log_2_ abundance (*center*), to TAMPOR log_2_ abundance (*right*). Yellow ovals in the center and right panels indicate the location of GIS samples in the MDS plot near the plot origin or center. **(D)** The population median of Pearson correlation coefficients (rho) modestly increases in TAMPOR versus naïve ratio (*left*), and in comparisons of the same replicate pair’s correlation coefficients with naïve ratio-based correction (blue) and after TAMPOR (red), *right*. Sixty two green segments connect pairs increasing in correlation, and 58 red segments connect ones decreasing in correlation. The absolute magnitude of the increases in rho was significantly greater than that of the decreasing population determined by a one-tailed *t*-test (*p* = 0.028). **(E)** Histogram of the population of Pearson rho values for naïve ratio non-replicate pairs (n = 28,560) among the 240 samples in the cohort for naïve ratio-corrected abundance (blue), and TAMPOR abundance (red, overlain transparent bars). Population means are shown above each dashed line at the mean Pearson rho for each population. The difference of means was significant (*p* < 2.2 × 10^−16^). **(F)** Change in Pearson rho (magenta bars) or bicor (deepviolet narrower bars) for the same pairs were plotted as a population histogram. The distribution of Pearson rho value differences is skewed positive, whereas the narrow distribution of differences in bicor values has no skew and centers at a mean of 0. **(G)** Volcano plot of the 769 proteins in naïve ratio-corrected proteomes of the 120 averaged sample replicate data. The green ellipse indicates proteins which shift substantially away from a large but insignificant positive log_2_ fold change in the data after TAMPOR correction. **(H)** Following TAMPOR, the volcano plot captures 17 more significant differentially abundant proteins. Inset, TAMPOR convergence plot shows the convergence criterion (green dashed line) was reached after 16 iterations.

This differs from Eq. 2 only by using GIS consistently in denominators of both ratio terms. We hypothesized that the more robust TAMPOR algorithm would improve correlation across pairs of technical replicate samples (Figure 4A).

TAMPOR here reduces variance gauged by the mean-SD trendline only marginally better than naïve ratio (Figure 4B), and the MDS plot of TAMPOR abundance is virtually identical to naïve ratio-normalized abundance, with GIS samples at the origin of the MDS plot of log_2_ (abundance) after either normalization (Figure 4C). Interestingly, median Pearson rho (R) correlation metrics for the 120 sample replicate pairs increased, albeit not significantly as a population mean of unpaired rho values (Figure 4D, left panel). However, comparison of the change in Pearson R across the pair of correlation coefficients from Abundance/GIS_intrabatch_ (blue points) to the corresponding pair correlation coefficient after TAMPOR (red points) showed that 62 of 120 pairs improved in correlation (green segments), and importantly, the mean absolute magnitude of change in those 62 paired R values (ΔRho_up_ = 0.010 ± 0.0028 SEM) was significantly greater than the magnitude of decrease in the other 58 pairs (ΔRho_down_ = 0.0043 ± 0.00060 SEM) (Figure 4D, right panel).

Furthermore, boosting of Pearson correlations was seen in the 28,560 R values for all unpaired sample correlations among the 240 samples, increasing the mean population R value from 0.020 to 0.11 (Figure 4E). In contrast, the same correlations calculated with robust biweight midcorrelation (bicor), which is less sensitive to outliers compared to Pearson correlation, showed relatively little change in correlation coefficient (ΔBicor) compared to the ΔRho population based on Pearson correlation coefficients (Figure 4F). This result suggests that sample-to-sample correlation structure of the data remains intact despite the boosting of Pearson correlations in the TAMPOR abundance data. Finally, the TAMPOR abundance data improved sensitivity for discerning differential abundance in replicate-averaged data, which represented N = 120 AD biomarker-positive and -negative case CSF proteomes, and where an additional 10 downregulated and 7 upregulated proteins were identified above nominal significance using TAMPOR abundance (Figure 4G vs. H). Interestingly, proteins not reaching significance but with a relatively large increase (>50%, or 0.58 log_2_-fold change) in AD vs. Control sample naïve ratio-derived abundance were not found in that area of the volcano plot of TAMPOR abundance (Figure 4G, green ellipse). These analyses suggest TAMPOR increases robustness and precision of measurements through its leveraging of the variance structure of both inter-protein and inter-sample abundance, where naïve ratio only considers inter-sample correction.

### Use case 4. Transposed TAMPOR harmonizes multi-platform proteomics abundance

**FIGURE 5:**
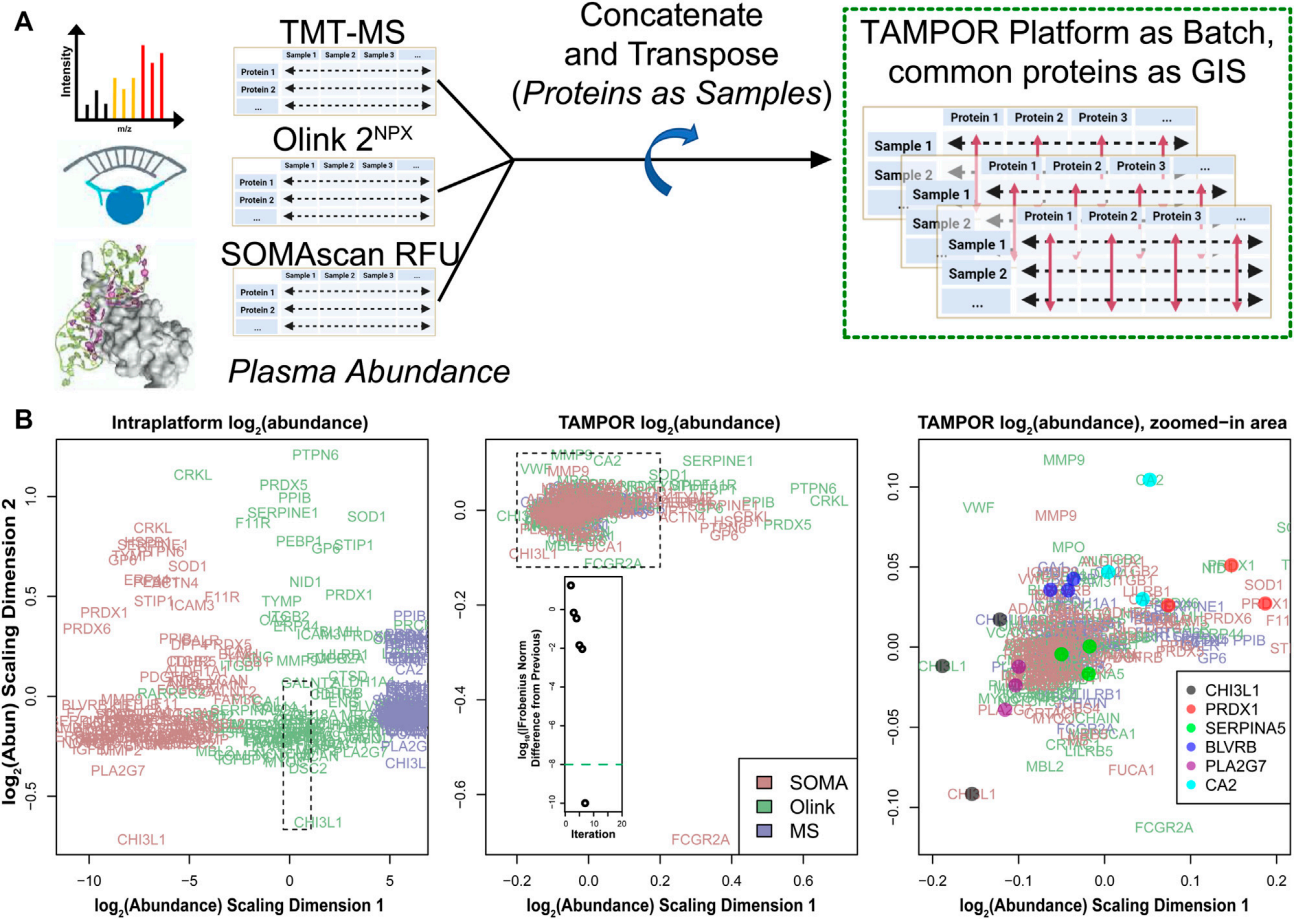
Use case 4: TAMPOR correction of multi-platform blood plasma proteomic data. **(A)** Protein measurements were collected on three platforms: TMT-MS, Olink, and SOMAScan, and minimally batch corrected within platform (i.e., using naïve ratio for TMT-MS, NPX normalization for Olink, and batch-corrected relative fluorescence units (RFU) for SOMAscan). The data were combined into a single matrix with strong batch effects due to platform, and the matrix transposed to place assays of protein isoforms as columns and samples as rows. 35 sample measurements were kept, replicated for the same samples on each of the platforms. None of the 9,057 protein assays had any missing values. **(B)** One hundred and one protein isoforms were common to all 3 platforms representing 101 gene product protein measurements across the 35 samples. A MDS plot of their variance indicates stark separation of the assay measurements by platform along the first dimension of separation in uncorrected log_2_ abundance (*left*). The dashed box indicates the border of the following plot. *Center*, after TAMPOR of the transposed data, the 303 assays have a focused, tightly overlapping distribution about a common central focus at the origin of the MDS plot. *Inset*, TAMPOR converged in 7 iterations. *Right*, further zooming into the area represented by the dashed box in the middle panel, and adding points for selected assay triplicates on the 3 different platforms for 6 separate protein isoforms quantified in the TAMPOR abundance matrix indicates close proximity of most triplets in the MDS plot space.

Protein abundance measurements from platforms other than MS, such as proximity extension assay (PEA) or aptamer-based affinity probes, are becoming more commonly used for discovery proteomics. In a pilot study where MS proteomics was performed on paired CSF and blood plasma from 36 AD and control elder individuals, 35 of the same individuals’ 2 biofluid proteomes were also profiled by the indirect measurement modalities of Olink (PEA, 13 panels of 92 analytes) and SOMAscan 7k (an aptamer-based platform). The data were analyzed independently with minimal normalization within platform before correlation of relative abundances measured across platform (Dammer et al., 2022). In this study, we also wanted to perform systems biology pipeline analysis on the full data from all 3 platforms. To assemble a harmonized dataset, we combined the abundances from all 3 platforms into a single matrix. For the plasma, the matrix represented 35 samples and 9,057 total protein isoforms assayed, considering only proteins with no missing values in the TMT-MS data. Across all 3 platforms there were 101 proteins measured with common Uniprot accessions. We chose to transpose the protein-sample matrix and then consider these 303 columns (out of 9,057) as a standard set of measurements with which to harmonize all protein abundances across the platforms, defining these as GIS and running TAMPOR on the transposed matrix with the minimally aggressive Eq. 3, and with the platform of each protein measurement defining batch (Figure 5A). To summarize, in this use case, proteins are treated as samples, common proteins measured on all three platforms are considered in place of GIS, and samples are handled in place of proteins (rows) for the input to the TAMPOR algorithm (using only GIS). Batches are defined as the different measurement platforms—SOMAscan, Olink, and mass spectrometry.

An MDS plot of variance for the 101 × 3 common protein assays before TAMPOR showed separation by platform along the first scaled log_2_ abundance dimension and over a wide range (Figure 5B, left panel). Following TAMPOR, which converged in 7 iterations, the same 303 protein assays had a new log_2_ abundance scaling dimension 1 with greatly reduced range (Figure 5B, middle panel; range is indicated by the dashed rectangle overlaid on the left panel). Zooming in on the focal cluster of proteins in this assay, it was possible to discern that many sets of the 3 replicate measurements for a single protein isoform as measured on each platform had similar variance across samples, judging by their proximity in the MDS plot following TAMPOR (Figure 5B, right panel; zoomed range is indicated by the dashed rectangle overlaid on the middle panel). The TAMPOR abundance for the full 9,057 assay plasma data harmonized across the 3 platforms was successfully analyzed for systems biology, with many of the 35 modules containing multiple platform measurements of the same protein, or related modules containing the replicate measurements. Network granularity (35 modules), and the biological coherence of modules enriching for biological processes suggested TAMPOR harmonization was appropriate for successful application of the co-expression systems biology pipeline (Dammer et al., 2022). This final use case demonstrates the versatility of TAMPOR to go beyond harmonization of abundance data within platform, to multiplatform −omics.

## Discussion

A necessary pre-requirement of systems biology analysis is having abundance data adjusted for batch effects. The use cases above demonstrate that batch is just one of multiple potential contributors to technical artefacts in -omics abundance data. TAMPOR borrows and improves upon the ratio-based correction commonly, but not exclusively, applied to correction of labeled mass spectrometry batch effects. We show here that TAMPOR is more versatile for use with multi-cohort, multi-platform -omics data, and even with data that has a GIS defect. Batches of samples are ideally designed with the forethought of balancing the biological traits in each batch, in which case the need for GIS is obviated. In the case when each batch has some common sample, or samples, which fit well into at least one biologically defined group, e.g., AD diagnosis or controls, although biological replicates in such a group may be heterogeneous, TAMPOR is designed to maintain the biological variance within the group(s) as highlighted with variance partition analysis. Thus, biological replicates may be used in place of GIS. TAMPOR in this case further maintains variance between samples within batch, minimizing variance across batches as explicitly defined in a categorical variable. The median, or central tendency, of selected or all samples defines the denominators of the TAMPOR equation depending on the choice of tuning, as illustrated by the 3 closely related sets of equations presented above. Our existing publications demonstrate that TAMPOR can be run sequentially to remove covariance with different categorical variables from data (Johnson et al., 2020; Johnson et al., 2022). This fits with an assumption that each variable’s contribution to variance is additive.

Here, we have demonstrated that TAMPOR-corrected abundance mitigates variance due to any categorical variables that can systematically hamper the detection of differentially abundant species. Further, we have observed boosting of Pearson correlation among technical replicates, which also occurs throughout comparisons of pairs of non-technical replicates, while having minimal effect on the median-based bicor across all the same sample pairs. We call this feature of TAMPOR “correlation boosting,” and Figure 4F suggests this occurs through an effect of Pearson correlations being brought into line with bicor *via* a tightening of the underlying correlation structure of the abundance matrix. We speculate correlation boosting by TAMPOR likely benefits downstream co-expression network and related systems analysis, so that sample connectivity is maximized in TAMPOR-corrected abundance. Notably, median polish approaches are robust to outliers, so that all samples for which there are data can be passed through TAMPOR. Rows with too much missing data, or noise-level data, on the other hand, would drag down sample (column) medians, however—so these are removed if greater than or equal to 50 percent of a row’s values, up front before the TAMPOR median polish. Typically, in a proteomics dataset with 10,000 to 12,000 data dependent acquired proteins, only 15 to 20 percent of rows are removed given the above criteria. That 60 to 70 percent of some RNA-Seq gene-products are likewise removed by filtering appropriately on noise level criteria reflects the amplified signals from which this data are obtained, owing to leaky transcription and other phenomena less applicable to mass spectrometry based measurements of the proteome.

After TAMPOR, outlier removal as well as other downstream cleanup of unwanted variance such as regression of age, sex, and/or postmortem interval can be applied. With TAMPOR, there is no need for a complex regression model for *a priori* removal of a categorical variable defining batches of samples (or measurement platforms), and protection of traits is unnecessary if batches were randomized with trait balancing (Maienschein-Cline et al., 2014). Outliers which would impact regression, requiring their up-front removal, can be left in during TAMPOR, defined by the MDS plot encompassing all variance of TAMPOR output in two dimensions (or by other approaches such as PCA, or coexpression network connectivity). We demonstrate that TAMPOR is able to adjust batches which contain outliers, e.g., Figure 2C, right panel, while focusing the variance of most proteins across non-outlier samples. As has been demonstrated in the work of John Tukey and others, median polish approaches are not sensitive to outliers, and this includes TAMPOR.

TAMPOR has been favorably compared to multiple other normalization methods for cleanup of tandem mass tag mass spectrometry (TMT-MS) data in head-to-head comparisons considering a single cohort (Weiner et al., 2022). For the merging of multicohort MS data, we highly recommend that peptide spectral match identification, peptide quantification, and rollup into protein quantitation is best redone on all cohorts’ raw data to enforce consistent parsimony of protein assembly from peptides across all cohorts. This does not mitigate inter-cohort batch effects, but it does eliminate an important and unnecessary confound. Using data searched with different protein rollup parsimony applied, even when the same proteomic database was used, equates to having short reads in RNA data aligned to different genomic scaffold versions in different cohorts and then trying to harmonize them. While TAMPOR is agnostic to the nature of the -omics input data, some considerations are warranted when applying the algorithm. These considerations are further discussed in the Appendix.

As more human cohort data become available in studies of AD, the brain, and in other fields, we foresee the benefit of having a means to harmonize the multi-omic abundance data for these cohorts to increase power of analyses. Harmonization allows for more direct comparison of abundance across different −omics, particularly transcriptomes and proteomes in health, aging, and disease. Systems biology analyses of AD focusing on co-expression modules and their biology are well powered with hundreds to thousands of samples. Recent studies have already achieved this power (McKenzie et al., 2017; Allen et al., 2018; De Jager et al., 2018; Yu et al., 2018; Johnson et al., 2020; Neff et al., 2021; Wingo A. P. et al., 2021; Wingo T. S. et al., 2021; Gandal et al., 2022; Johnson et al., 2022; Wingo et al., 2022). Once samples can be harmonized in the ten to hundreds of thousands, with thousands of quantified gene products in each sample, additional analyses leveraging machine learning become feasible. We anticipate TAMPOR or related techniques for harmonization of -omics data will be a useful tool enabling such future work tomorrow, while improving batch correction today.

## Data Availability

Data for use cases in this study are available at https://www.synapse.org/DeepConsensus (Use case 1), https://www.synapse.org/Consensus (Use case 2), https://www.synapse.org/#!Synapse:syn20821165 and ProteomeXchange resource PXD009589 accessible via https://ebi.ac.uk/pride (Use case 3), and https://www.synapse.org/3platformEmory (Use case 4). The TAMPOR algorithm is implemented in R and available on GitHub at https://www.github.com/edammer/TAMPOR.

## Appendix: Consideration of −Omics Abundance Data and Characteristics of TAMPOR Input

TAMPOR was designed to meet the challenge of delivering an -omics abundance data cleanup method which is versatile in its application regardless of acquisition characteristics, and which can optionally leverage standard sample replicates across batched data, but being robust and adaptable enough to work on abundance data acquired in any possible way. An important consideration in analyzing and cleanup of proteomics datasets historically has been *how the data were acquired*, impacting missing values in the data and often setting a prior requirement for inclusion of standard sample replicates across batches of samples. Standard samples are often prepared as equal mixture pools of all individual samples in a cohort and inserted into sample sets which are run as a batch, but their use is far from universal, especially outside of TMT-MS based proteomics. The inspiration for TAMPOR comes from ratio-based normalization of TMT based workflows with different batches of samples that each contain at least one standard sample replicate. This section highlights the complexities and implications of -omics abundance data acquisition impacting its analysis and cleanup.

In mass spectrometry (MS), label-free quantitation (LFQ) has different aspects of within- and across-batch variance compared to isobaric label-based quantitation such as tandem mass tag mass spectrometry (TMT). Both MS methods rely on data-dependent acquisition (DDA), in which the highest signal precursors in a protein mixture are selected for fragmentation and used to generate quantitative data after identification of peptides from their fragmentation pattern in the spectrometer, which leads to missing data when a protein detected and quantified in one injection into the mass spectrometer is not detected or quantified in another injection. Missingness of data in LFQ and TMT DDA data differs in that a single sample will miss protein quantitation and even identification in LFQ, but a whole TMT batch usually loses quantitation of a missed protein. If missing data were imputed, assumptions of imputation of data as missing completely at random are not accurate, and one should be more cautious of imputing TMT batchwise missing data. Thus, TAMPOR is not typically run using data with missing values imputed, though this practice could be explored in the future. Different depth of coverage in terms of the number of proteins assayed by any LFQ- vs. a TMT-quantified cohort also must cause one to approach the use of TAMPOR for harmonizing TMT and LFQ with caution and some foresight in preparation of the data before merging for obtaining TAMPOR abundance.

Another approach to acquire MS data is through data independent acquisition (DIA), in which ions within a certain mass-to-charge (m/z) window are fragmented to produce multiplex MS/MS peptide fragmentation spectra that must be deconvoluted and assigned to their respective precursor ions’ quantitative signals. DIA produces raw data with unique characteristics that require different software algorithms for extraction of quantitative peptide-level information. The abundances which result from this extraction are subject to effects of ion interference in any crowded scan from which quantitation occurs, whether a precursor scan (MS) or MS/MS (i.e., MS2)—though DIA quantitation is sampling from much higher signal:noise in a precursor scan (Müller et al., 2019). Quantitation of DDA TMT reporter ions can also suffer from contaminating noise in the reported signal, which is improved in one strategy leveraging MS3-based quantitation of the reporter, at the expense of machine time and depth of coverage of the proteome (Erickson et al., 2015).

Several advances in mass spectrometer hardware and software have been introduced which also can affect less interference in complex samples, thereby causing different levels of compression of differential protein abundance quantitation compared to older platforms. The 50 batches of ROSMAP data we presented in use case 1 here mostly implemented MS2-based TMT reporter quantitation, but ∼10% of batches used MS3-based quantitation. Notably, ROSMAP TAMPOR abundance of the 400 samples was not systematically biased by the difference in compression of fold change which exists in the underlying raw data, because systems biology analysis showed that samples segregated by their pathology and extent of AD (asymptomatic vs. symptomatic AD vs. non-impaired individuals with little or no pathology), and not the mode of TMT quantitation in the mass spectrometer (Johnson et al., 2020; Johnson et al., 2022). This suggests that TAMPOR is robust to compression effects and can harmonize disparate cohorts with different compression of fold change, though further exploration of this using simulated data is warranted.

Affinity-based proteomics methods such as those commercialized by Olink and SomaLogic have their own data characteristics, with measurements that can be below limit of detection or failing internal quality control, but without explicitly missing data. Different signal:noise in these indirect platform measurements is a concern, but is addressable and we show here in use case 4, and in a pilot study performing coexpression analysis on TAMPOR abundance of both Olink and SOMAscan harmonized with TMT-MS that the multi-platform TAMPOR abundance is again robust to prior platform effects (Dammer et al., 2022).

As mentioned, bottom-up “shotgun” or “discovery” DDA proteomics data are prone to missing values, especially with larger cohorts. However, this aspect of the data comes with an advantage: the distribution of log-transformed abundances is generally unimodal to begin with because it derives from sampling only what produces above-noise signals of precursor ions in an MS scan for MS/MS fragmentation, and then further requires acceptable signal:noise of fragment ions to score and identify peptide sequences. This signal:noise threshold is commonly set to 10:1 in Proteome Discoverer (PD)’s default settings (ThermoFisher, 2017). To what extent normality (a proper Gaussian distribution) of abundance data input to TAMPOR is important is open to further exploration. However, next-generation RNA sequencing abundance matrices pose an extreme example of why this is relevant. In contrast to proteomics which in most cases has limited capacity for amplification of the physical substrate, RNA-seq or microarray abundance data has no missing values, but there is noise at the left tail of the log (abundance) distribution. Thus, raw transcriptomic data often has a bimodal distribution of log_2_ (abundance). To make such data amenable to TAMPOR, the rows of data can be censored, treating 0 and low signal (mostly noise) values as missing data and setting a maximum threshold for each row representing gene products that are well expressed in the biological matrix, and removing ones that are mostly not expressed above noise (Dill et al., 2021). Following censoring, transcriptomic data are amenable to the same correction approaches as proteomic data.
